# Days to visit an offshore island: effect of weather conditions on arrival fuel load and potential flight range for common blackbirds *Turdus merula* migrating over the North Sea

**DOI:** 10.1186/s40462-021-00290-6

**Published:** 2021-10-21

**Authors:** Natalie A. Kelsey, Ommo Hüppop, Franz Bairlein

**Affiliations:** 1 Institute of Avian Research ‘Vogelwarte Helgoland’, An der Vogelwarte 21, 26386 Wilhelmshaven, Germany; 2 Max Planck Institute of Animal Behavior, Am Obstberg 1, 78315 Radolfzell, Germany

**Keywords:** Arrival fuel load, Body condition, EchoMRI, Environmental condition, Flight ranges, Flow-assistance, Migratory strategy, Quantitative magnetic resonance, Offshore bird migration, Weather conditions

## Abstract

**Background:**

Crossing open water instead of following the coast(line) is one way for landbirds to continue migration. However, depending on prevailing weather and the birds’ physiological conditions, it is also a risky choice. To date, the question remains as to which interplay between environmental and physiological conditions force landbirds to stop on remote islands. We hypothesise that unfavourable winds affect lean birds with low energy resources, while poor visibility affects all birds regardless of their fuel loads.

**Methods:**

To test this hypothesis, we caught 1312 common blackbirds *Turdus merula* stopping over on Helgoland during autumn and spring migration. Arrival fuel load was measured using quantitative magnetic resonance technology. Weather parameters (wind and relative humidity as a proxy for visibility) were interpolated for the night before arrival. Further, we calculated whether caught individuals would have successfully crossed the North Sea instead of landing on Helgoland, depending on wind conditions.

**Results:**

Both wind and relative humidity the night before arrival were correlated with arrival fuel load. After nights with strong headwinds, birds caught the following day were mostly lean, most of which would not have managed to cross the sea if they had not stopped on Helgoland. In contrast, fat birds that could have successfully travelled on were caught mainly after nights with high relative humidity (≥ 80%). Furthermore, the rate of presumably successful flights was lower due to wind: although only 9% of all blackbirds captured on Helgoland had insufficient fuel loads to allow safe onward migration in still air, real wind conditions would have prevented 30% of birds from successfully crossing the sea during autumn and 21% during spring migration.

**Conclusions:**

We were able to decipher how physiological condition, wind and relative humidity partially force blackbirds to stop on a remote island. Adverse winds tend to affect lean birds with low energy resources, while poor visibility can affect blackbirds, regardless of whether the arrival fuel load was sufficient for onward flight. Our findings will help to understand different migratory strategies and explain further questions like migration timing.

**Supplementary Information:**

The online version contains supplementary material available at 10.1186/s40462-021-00290-6.

## Background

During migration, birds are found in large numbers in coastal stopover areas [1]. From there, crossing the sea is often one possibility to continue migration, providing advantages such as a significant reduction of flight distance and time spent on migration [2, 3]. But, depending on prevailing weather conditions, it is also a risky choice with unique challenges, especially for terrestrial birds, as it covers a large, inhospitable area with limited opportunities to rest and refuel [4, 5].

Depending on the migratory strategy used, the birds’ physiological conditions (mainly fuel load) and environmental conditions, the open sea may even act as an ecological barrier [5–7]. Crossing the open sea can bear a high mortality risk if landbirds make navigational errors, deplete their fuel loads too early and/or face sudden unfavourable weather conditions [8, 9]. The latter can lead to poor or deteriorating flight conditions and even hinder the continuation of migration by causing “Zugstau”, i.e. local accumulation of migrants in a certain area due to a weather-related interruption of migration [10–12]. On the other hand, successful crossing of a barrier is closely related to supportive weather conditions [4–6].

These weather conditions together with the bird’s fuel load lead to a variety of observed strategies when birds are confronted with such barriers, i.e. crossing or circumventing [13, 14]. Further, weather conditions have been shown to shape, e.g., migratory timing [15–18] and the bird’s departure decision from the stopover site [16, 19–21]. Weather conditions influencing the individuals’ migratory decision include temperature [19, 21, 22], precipitation [16–18] and cloud cover [21–24]. However, the most influential weather covariates are pressure changes, wind and relative humidity (among others as a proxy for visibility) [7, 12, 20, 24]. Wind support is one of the most interesting, as it affects flight duration and range, the birds’ orientations and groundspeeds [25, 26]. It also indirectly influences the birds’ fuel reserves: strong head- or crosswinds increase fuel consumption [27], while greater wind support minimises travel time and fuel expenditure [28, 29].

While crossing the sea, islands provide the only natural opportunity for landbirds to rest, refuel and escape possible unfavourable weather conditions before successfully reaching the coast. Especially offshore islands like the small island of Helgoland [30], located in the North Sea about 45 km from the nearest island or coast, frequently exert a strong attraction on large numbers of migrants [10, 11, 31, 32]. The North Sea represents a possible but small ecological barrier for bird migration along the East Atlantic flyway [10, 33] because wind conditions in autumn are usually unfavourable for birds’ migrating south-west with predominantly westerly and south-westerly winds experienced as head- or crosswinds [23, 34]. In contrast, prevailing westerly winds in spring have a supportive direction for migrants [35, 36]. The number of birds stopping on Helgoland is influenced by these weather conditions at the respective site: in good weather, only a small proportion of migrants stop on Helgoland, while most birds continue [37]. In deteriorating weather conditions, on the other hand, the number of birds “forced” to land, e.g., by the onset of fog or drizzle and/or drifted by wind, increases sharply [10, 30]. To what extent dwindling energy reserves force birds to interrupt their journey is little known, especially in combination with weather conditions. Generally, fuel load is not considered a strong factor forcing birds to land on Helgoland, as their further journey across the sea, especially in autumn, is comparatively short, seems to require only low or moderate fuel loads and is therefore not considered a major problem for migrating birds [10, 38]. But it is still unclear how lean and/or fat birds are affected when they choose not to stop on Helgoland but to travel on even though weather conditions are not conducive.

Understanding these (local) environmental and intrinsic arrival conditions that lead to the decision to interrupt flight is crucial for understanding birds’ movement and for theoretical predictions regarding different migratory strategies [39]. Yet, to our knowledge, it is still not fully understood how these conditions actually force birds to stop on a remote island like Helgoland. We hypothesise that unfavourable winds rather pressure lean birds with low energy resources to land, while poor visibility generally forces birds to land, regardless of the sufficiency of their arrival fuel load.

To investigate this, we measured the arrival fuel load (using quantitative magnetic resonance technology; QMR) of a nocturnal short-distance migrant, the common blackbird *Turdus merula* (hereafter “blackbirds”), one of the most frequent species stopping on Helgoland while crossing the North Sea during migration. We then analysed the influence of local wind and relative humidity—two of the most important weather parameters effecting migratory decisions during spring and autumn migration [15, 39, 40]—on the blackbirds’ arrival fuel loads. Additionally, we included other intrinsic factors that may influence birds’ response to weather conditions [41], i.e. age (adult experience [42, 43]), sex (males reach breeding grounds earlier to establish prime territories [3, 44]) and season (time pressure to reach breeding grounds in spring [45]). However, we assume that these factors are rather secondary compared to weather influences.

## Methods

### Bird data

**Fig. 1:**
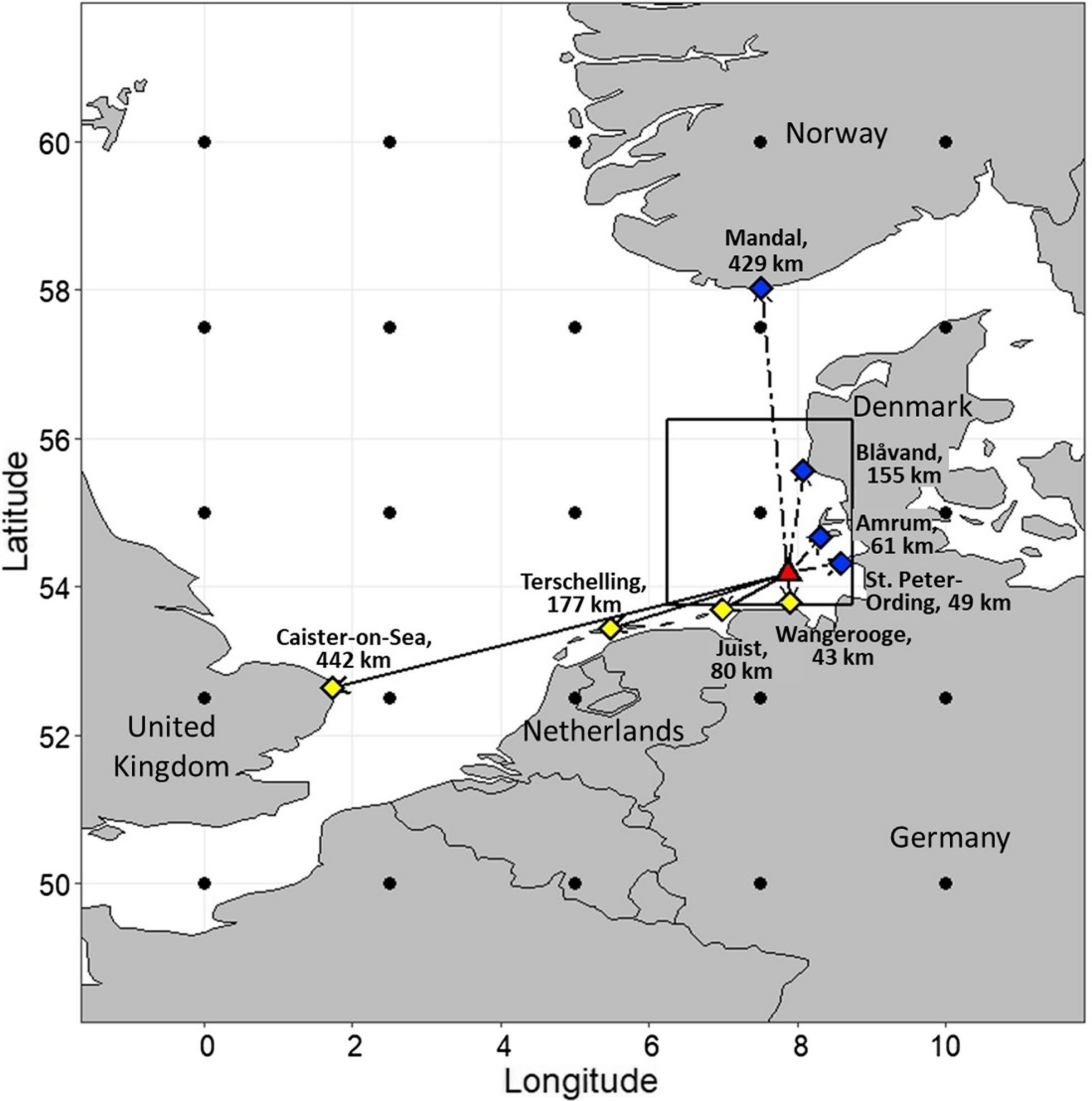
The study area is Helgoland (red triangle). Based on ringing recoveries, four possible coastal destinations for autumn (yellow diamonds) and spring (blue diamonds) were chosen as example flight paths (autumn = solid lines; spring = dashed lines) with different flight distances (km). The area covered from 50° to 60°N and 0° to 10°E is subdivided by a spatial resolution of 25 NCEP reanalysis grid cell intercepts (2.5° × 2.5°); exemplified by Helgoland (solid box). Black dots represent the centre of each grid from which the weather parameters are obtained (see methods)

1312 blackbirds of both sexes and age classes were caught, ringed and measured (cf. [10]) in the trapping garden of the Institute of Avian Research on Helgoland (54.18°N, 7.88°E) during autumn and spring migration 2017–2019 (Fig. 1). These birds breed mainly in southern Fennoscandia and travel via the East-Atlantic flyway to reach their wintering grounds, particularly in the UK and western France [30, 46]. A flowchart for a better understanding of the following overall methods, can be found as Additional file 1: Fig. S1 (Additional file 1: chapter 1).

Body mass (± 0.1 g), using an electronic scale, and the QMR fat mass (± 0.01 g; hereafter: “absolute fuel load”), using the EchoMRI™ (EchoMRI Body Composition Analyser E26-262-BH, Zinsser Analytic GmbH, Frankfurt am Main, Germany), were measured for each bird on the day of first capture. This is considered to be the day of arrival [21], as most ringed blackbirds only stay on Helgoland for one or two days [47] with a median of four to five days in radio-telemetered blackbirds [21, 48]. Furthermore, it can be assumed that birds are most mobile on the first day after arrival and therefore the probability of capture is highest [49–51]. Sex and age (latter only during autumn: “first-years”, i.e. first-calendar year birds, and “adults”, i.e. birds older than first-calendar year) of the blackbirds were classified based on plumage characteristics [52]. After measuring and ringing, the birds were released.

The EchoMRI™ distinguishes between the different hydrogen spin relaxation rates of different body tissues and fluids in small animals up to a standard mass limit of 500 g [53]). This method provides precise, accurate and repeatable measurements of the avian body composition [54, 55]. Following Kelsey and Bairlein [55], we scanned each bird three consecutive times to allow for individual mean estimates. For more details see Kelsey and Bairlein [55], Kelsey et al. [56] and Additional file 1: chapter 2.

We estimated the individuals’ lean body mass by subtracting the absolute fuel load from the actual body mass. We then divided the absolute fuel load by the estimated lean body mass to calculate the birds’ relative fuel load (hereafter “arrival fuel load”).

### Weather data and tailwind components

In the following sections, all R-functions are part of the package “RNCEP” [57]. Spatiotemporal weather data were obtained using the National Centers for Environmental Prediction (NCEP) Reanalysis I dataset from the National Oceanic and Atmospheric Administration (NOAA/OAR/ESRL PSD, Boulder, Co, USA [58]) via the R-function “NCEP.gather”. The extracted spatial grid covers Helgoland (nearest weather data point: 55°N and 7.5°) and the surrounding area from 50° to 60°N and 0° to 10°E with a spatial resolution of a grid cell with 2.5° × 2.5°, resulting in 25 grid cell intercepts along the covered area (Fig. 1). For each grid, the relative humidity (“rhum”; %) as well as u- and v-wind components (“uwnd”: east/west, “vwnd”: north/south; m/s) of each capture day during spring (1^st^ March to 18^th^ May) and autumn migration (5^th^ October to 27^th^ November) 2017–2019 were used. These parameters were obtained for the pressure levels 1000 (near surface), 925 (~ 760 m above sea level) and 850 (~ 1500 m) hPa at 00:00 UTC (Additional file 1: Table S1, Additional file 1: chapter 3; including other not considered weather parameters), as these represent the most likely migration altitudes [35, 59] and are temporally close to the peak of the nocturnal migration after midnight [12, 21].

We used the u- and v-wind components to calculate the pressure level-dependent tailwind components (hereafter TWC [m/s]; Additional file 1: Table S1) for each individual by using the R-function “NCEP.Airspeed”, which requires u- and v-wind components as well as the assumed airspeed and preferred flight directions of the bird. For our blackbirds, we assumed an airspeed of 10 m/s [60] and different flight directions depending on the season and oriented towards the selected potential flight paths (explained in the following chapter). Negative TWC values describe obstructive winds (i.e. headwinds) and positive values assisting winds (i.e. tailwinds).

As migratory birds are most likely to “choose” the appropriate flight altitude to seek more supportive winds [28, 29, 35], increasing survival probability when crossing an ecological barrier [6, 61], we had NCEP.Airspeed select the flight altitude (including associated relative humidity values) with the highest TWC for each individual (Additional file 1: Table S2, Additional file 1: chapter 3). If more than one flight altitude had the highest value, the lower altitude of these was selected, as birds generally concentrate around the lowest altitude with supportive winds [35]. These altitude-dependent weather conditions were selected for each of the flight paths aligned with the selected coastal destinations (explained in the following chapter).

### Flight distance and direction

For blackbirds stopping on Helgoland, four different exemplary coastal destinations with increasing flight distances (km) and different flight directions (°), which were determined by the R-function “NCEP.flight”, were defined for each season (Fig. 1; Additional file 1: Table S3, Additional file 1: chapter 4). Coastal destinations were selected depending on their location along the coastline along the blackbirds’ assigned migration route, based on descriptions in the avifauna of Helgoland [30] as well as in the ringing atlases of Germany [62], Norway [63] and Sweden [64]. In autumn, we selected the coastal destinations Wangerooge (Germany), Juist (Germany), Terschelling (Netherlands) and Caister-on-Sea (United Kingdom), while in spring, we chose St. Peter-Ording (Germany), Amrum (Germany), Blåvand (Denmark) and Mandal (Norway).

### Successful flights (individual flight ranges and required fuel load)

As we wanted to identify possible causes for the blackbirds’ stopover on Helgoland, we calculated whether caught individuals would have managed the required flight distances to cross the North Sea to the coastal destinations instead of landing on Helgoland. This individual flight range depends on the experienced wind conditions corresponding to the different coastal destinations and the individual’s arrival fuel load. Therefore, we 1) simulated flight trajectories and 2) calculated the required fuel load.

With point 1), we determined on which observation days the wind conditions along the possible flight paths would allow birds to reach the respective coastal destinations. For this purpose, we used the R-function “NCEP.flight”: This function simulates single flight trajectories according to the specified behavioural rules and thus determines how the animal will behave and move in relation to the flow-assistance (i.e. wind support, considering TWC and cross winds [65]). The simulation was set to start at midnight (see above) on Helgoland and end locations were defined as each selected coastal destination. After the start of the simulation, the altitude from which flow-assistance was to be obtained (1000, 925, 850 hPa) was automatically recalibrated every half hour. The simulations stopped when 1) the final destination was reached, 2) flow-assistance fell below -12 m/s or 3) simulated flights ran for 18 h; the first case means that the model bird reached the final destination, the last two cases mean that it did not reach the destination. For the successful cases, NCEP.flight estimated the flight duration.

This estimate was used in point 2) to calculate the fuel load required for a successful crossing of the sea on each individual day. For this, we used equation two from Delingat et al. [66]: flight duration [h] = 100 × ln(1 + arrival fuel load) and rearranged:

1 $${\text{Required}}\,{\text{fuel}}\,{\text{load}}\,[{\text{rel}}.] = e^{{({\text{flight}}\,{\text{duration [}}h]/100)}} {-}1$$

We defined a bird as successful in reaching a coastal destination (hereafter “successful flight”) when the bird travelled on days with supportive flow-assistance (point 1) and the bird’s arrival fuel load was greater than the required fuel load (point 2).

In order to disentangle the effects of wind and birds’ fuel loads, we additionally investigated whether the birds already had sufficient fuel loads at arrival that would theoretically allow them to reach the coastal destinations under still air (zero flow-assistance). To do this, we used equation three from Delingat et al. [66]: flight range [km] = 100 × airspeed [km/h] × ln(1 + arrival fuel load) [h], where airspeed is the birds’ own speed in still air. This equation was then rearranged again:

2 $${\text{Sufficient}}\,{\text{fuel}}\,{\text{load}}\,({\text{rel}}.) = e^{{({\text{flight}}\,{\text{range}}\,[{\text{km}}]/(100 \times {\text{airspeed}}[{\text{km}}/{\text{h}}])][{\text{h}}]}} {-} \, 1$$

where the flight range corresponds to the various coastal destinations (Fig. 1) and the airspeed was 10 m/s (= 36 km/h), as described above (overview of average fuel-dependent flight range, including fat score levels: Additional file 1: Table S4, Additional file 1: chapter 5).

### Statistical analyses

We conducted the data analyses using R [67] and provided uncertainty measures (2.5% and 97.5% quantiles of the symmetric 95% credible intervals, CrI) for each model using the function “sim” from the R-package “arm” (cf. [68]). An effect was specified as strong if the associated CrI did not include zero or did not overlap between comparing groups.

Differences in body mass, lean body mass and absolute fuel load (dependent variables) between birds of different sex (two-level explanatory factor: male and female) or age (first-years and adults) or between the migratory seasons (autumn and spring) were tested with linear models (LM), including all corresponding interactions between the explanatory variables.

Since the arrival fuel load (dependent variable) is given in relative proportions, we used a generalised linear model with a binomial error distribution (GLM; family “quasibinomial”) to test for effects of sex, age and season (explanatory variables). The GLM family “quasibinomial” transforms the original data with a logit function (= logit^−1^(x)). To compare the original arrival fuel load values between the groups, we back-transformed the data with the inverse logit function (= e^x/(1+ex)^) provided by the R-function “plogis”.

The models used to analyse the relationship between the birds’ arrival fuel load and altitude-dependent weather conditions (see above) also consisted of GLMs separately for each season. TWC and relative humidity were included as continuous explanatory variables, sex and age (including the two-way interaction in autumn) as two-level explanatory factors, and arrival fuel load as dependent variable. For relative humidity, we used orthogonal polynomials up to a quadratic degree, which are uncorrelated and, therefore, allow the estimated linear and quadratic effects to be interpreted as purely linear and purely quadratic influences of the predictor on the outcome [68]. TWC was only included for a linear regression, as quadratic regressions were never significant in initial models.

As TWC depended on the assumed flight direction, for which we had chosen different possibilities, we calculated models separately for each of the flight paths oriented towards the coastal destinations in spring and autumn. Using the function “vif” from the R-package “car” [41], all continuous explanatory variables of our models were found to be non-collinear (variance inflation factor < 2) [69]. While GLM-transformed data are provided to compare effects between the explanatory variables and arrival fuel load, back-transformed data were used to visualise the results.

In addition, we investigated arrival fuel load between migrants caught after “unfavourable weather” nights and migrants caught otherwise (after “favourable weather” nights). We defined unfavourable weather nights as either nights with TWC ≤ -5 m/s (following Erni et al. [39]; hereafter “adverse winds”) and/or a relative humidity ≥ 80% (“high” relative humidity; hereafter “h-rhum”), as the latter is closely associated with poor visibility and orientation due to heavy cloud cover, fog, drizzle and precipitation [12, 70, 71]. Favourable weather nights included TWC > -5 m/s (hereafter “favourable winds”) and/or relative humidity < 80% (“normal” relative humidity; hereafter “n-rhum”). The arrival fuel loads (dependent variable) of the individual blackbirds were compared between favourable and unfavourable weather nights as two-level explanatory factors (relative humidity: n- and h-rhum; TWC: favourable and adverse winds) using GLMs. Again, the models were considered separately for both seasons and flight paths aligned with the coastal destinations, and model outputs provided here include back-transformed values to allow comparisons of group means.

Further parameters and interactions initially tested but excluded from further analyses, can be found in Additional file 1: chapter 6). We assessed model assumptions (e.g. normal distribution of residuals, Tukey-Anscombe Plot) according to Korner-Nievergelt et al. [68]. Inspections of residuals plots presented no violation of the model assumptions for any model.

## Results

### Percentage distribution and group comparison of arrival fuel load

**Fig. 2:**
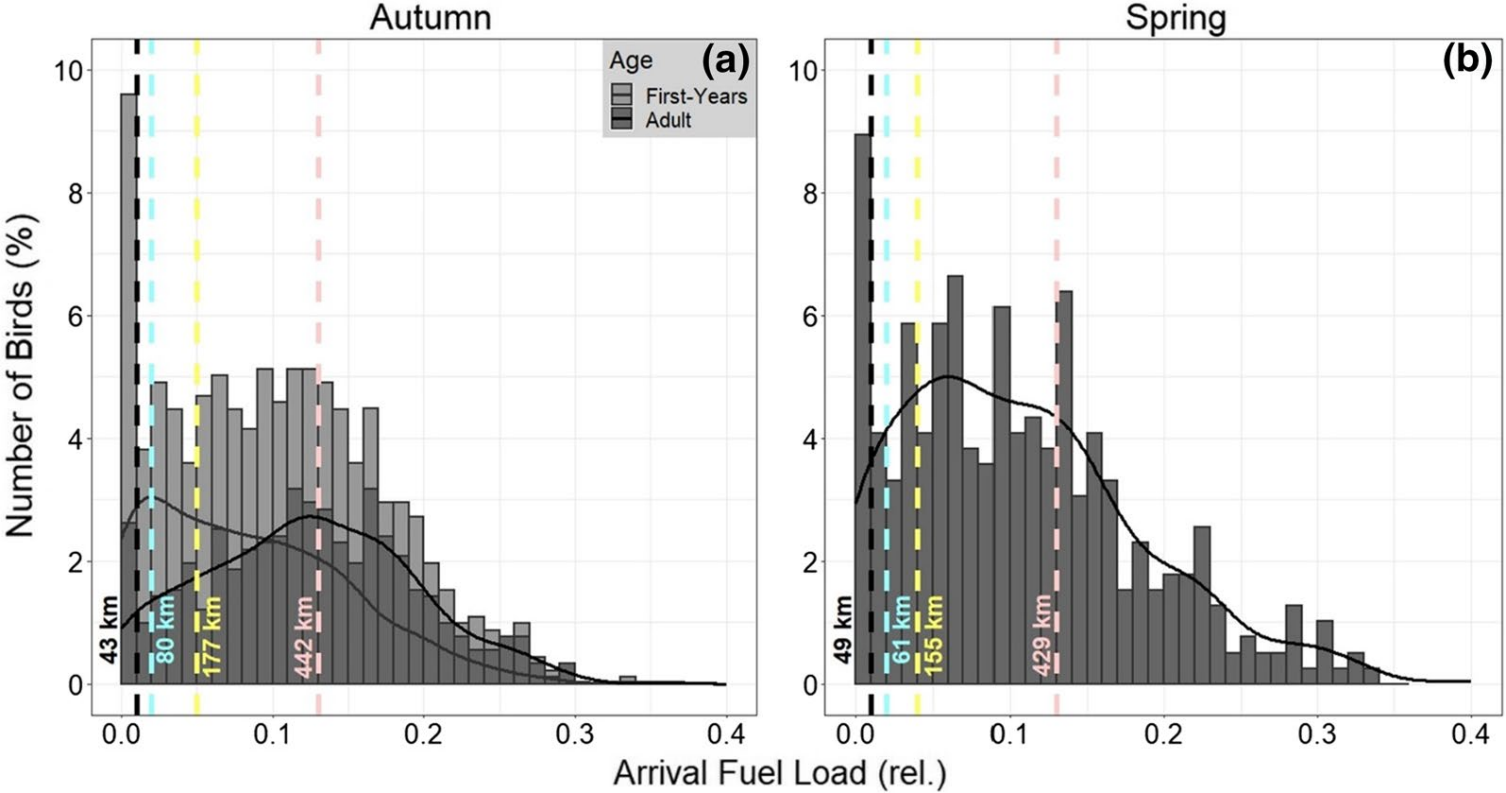
Arrival fuel loads (relative values) of migrating blackbirds caught on Helgoland during a) autumn (n = 919) and b) spring migration (n = 393). Age differentiation is only possible in autumn (first-years n = 461; adults n = 458). The curved lines represent the kernel density estimates, i.e. the smoothed version of the histograms. The dashed lines describe the fuel loads needed to reach the different coastal destinations (Fig. 1) in still air (zero flow-assistance): **a** from left to right Wangerooge, Juist, Terschelling and Caister-on-Sea; **b** St. Peter-Ording, Amrum, Blåvand and Mandal

In both seasons, 91% of the blackbirds studied (autumn: n = 919; spring: n = 393) already carried sufficient fuel loads (≥ 1%) on arrival that would theoretically allow them to continue their journey immediately and reach the nearest coastal destination in still air (autumn: Wangerooge; spring: St. Peter-Ording; Fig. 2). In 36% of the birds in both seasons, we even found sufficient fuel loads (≥ 13%) to allow them to continue their migration over sea and reach the furthest coastal destination (autumn: Caister-on-Sea; spring: Mandal).

In autumn, where we were able to compare age classes, we found a lower average arrival fuel load (Table 1) and, thus, lower rates of successful flights (Additional file 1: Table S5, Additional file 1: chapter 6), for first-year birds than for adults in both sexes. Sexes did not differ, neither in first-year birds nor in adults. On the contrary, in spring males were on average fatter and had higher successful flight rates than females.

**Table 1:** Average body mass, lean body mass, absolute and relative arrival fuel load

Season	Sex	Age	n	Body mass (g)	Lean body mass (g)	Absolute fuel load (g)	Arrival fuel load (rel.)
Autumn	Male	First-years	212	98.1 (96.9–99.3)	90.61 (89.85–91.40)	7.47 (6.62–8.30)	0.082 (0.073–0.091)
		Adults	219	105.7 (104.5–106.9)	93.26 (92.45–94.01)	12.45 (11.59–13.25)	0.134 (0.124–0.145)
	Female	First-years	249	95.6 (94.5–96.7)	87.83 (87.13–88.56)	7.73 (6.94–8.50)	0.088 (0.080–0.096)
		Adults	239	100.4 (99.2–101.5)	89.98 (89.24–90.70)	10.36 (9.56–11.14)	0.115 (0.106–0.125)
Spring	Male	Adults	200	102.8 (101.5–104.0)	91.65 (90.82–92.43)	11.12 (10.19–11.97)	0.121 (0.111–0.132)
	Female	Adults	193	95.0 (93.7–96.3)	86.79 (85.98–87.65)	8.22 (7.35–9.18)	0.095 (0.085–0.104)

Data are given for all migrating blackbirds (n = 1312), broken down by sex, age and season. For each group, sample size (n), mean values and 95% credible intervals (2.5% and 97.5%) are reported

### Arrival fuel load and weather

**Fig. 3:**
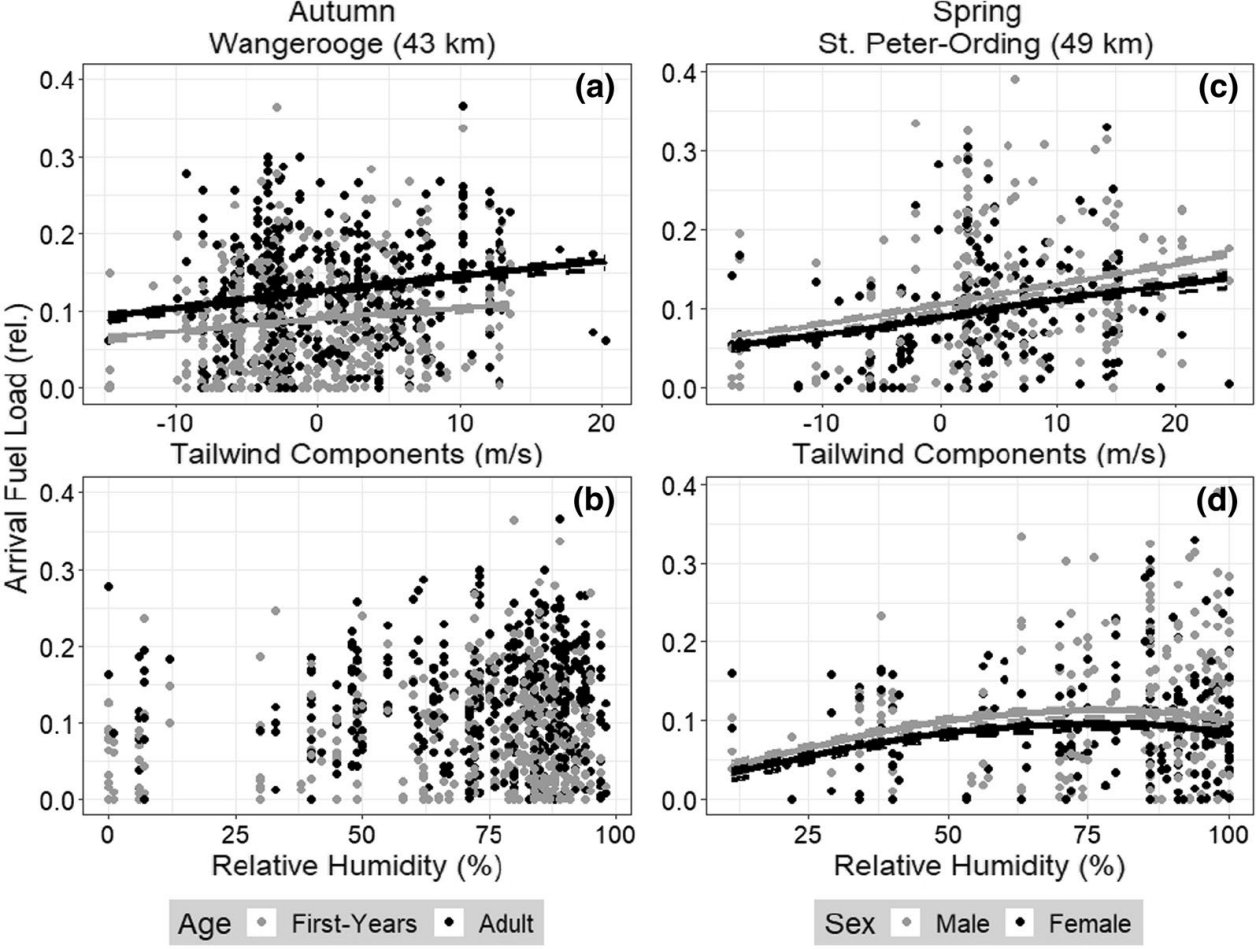
Blackbirds’ arrival fuel load (relative values) regressed against tailwind components (TWC, m/s; **a**, **c**) and relative humidity (%; **b**, **d**) experienced the night before arrival. For the weather data shown here, the values given are dependent on TWC calculated for flight direction towards the assumed coastal destinations of Wangerooge (autumn; 43 km flight distance from Helgoland) and St. Peter-Ording (spring; 49 km). Data is broken down by age (grey = first-years; black = adults) and sex (grey = males; black = females) for autumn (n = 919; **a**, **b**) and spring (n = 393; **c**, **d**)

TWC, calculated for the flight path aligned with the nearest coastal destination Wangerooge during autumn migration and with St. Peter-Ording and Amrum during spring migration (Fig. 1), showed a positive regression with arrival fuel load (Table 2, Fig. 3a,c), i.e. the lower the TWC during the night, the lower the arrival fuel load of birds caught on Helgoland the following day. Relative humidity was correlated with arrival fuel load (positive linear and/or positive or negative quadratic regressions; Table 2, Fig. 3d), with the exception of the flight path that was directed towards Wangerooge during autumn migration (Fig. 3b). Age, sex and the interaction, i.e. that the effect of sex can change with age and vice versa, influenced arrival fuel load in all models.

**Table 2:** Correlation between arrival fuel load, tailwind components (TWC), relative humidity, sex and age. Separate models were run for both seasons, with each flight path aligned with a coastal destination (Fig. 1)

Autumn migration	Wangerooge (43 km)	Juist (80 km)	Terschelling (177 km)	Caister-on-Sea (442 km)
Intercept	**−** **2.415 (−** **2.521 to −** **2.302)**	**−** **2.405 (−** **2.517 to − 2.301)**	**− 2.411 (− 2.523 to − 2.307)**	**− 2.413 (− 2.159 to − 2.307)**
Tailwind components (linear)	**0.017 (0.009**–**0.025)**	0.003 (− 0.006 to 0.012)	− 0.004 (− 0.012 to 0.005)	− 0.006 (− 0.014 to 0.003)
Relative Humidity (linear)	1.499 (− 0.104 to 3.215)	**2.136 (0.592**–**3.630)**	**1.887 (0.359**–**3.516)**	**1.832(0.290**–**3.474)**
Relative Humidity (quadratic)	− 0.526 (− 2.089 to 1.033)	**1.600 (0.031**–**3.080)**	**1.512 (0.064**–**3.046)**	1.445 (− 0.013 to 2.971)
Sex	0.078 (− 0.066 to 0.222)	0.071 (− 0.070 to 0.214)	0.073 (− 0.068 to 0.215)	0.073 (− 0.069 to 0.216)
Age	**0.517 (0.379**–**0.654)**	**0.532 (0.393**–**0.672)**	**0.522 (0.385**–**0.661)**	**0.517(0.377**–**0.658)**
Interaction (Sex:Age)	**− 0.229 (− 0.417 to − 0.034)**	**− 0.230 (− 0.419 to − 0.036)**	**− 0.231 (− 0.420 to − 0.043)**	**− 0.227 (− 0.417 to − 0.036)**

Spring migration	St. Peter-Ording (49 km)	Amrum (61 km)	Blåvand (155 km)	Mandal (429 km)
Intercept	**− 2.199 (− 2.236 to − 2.004)**	**− 2.042 (− 2.157 to − 1.930)**	**− 1.985 (− 2.094 to − 1.879)**	**− 1.970 (− 2.077 to − 1.866)**
Tailwind components (linear)	**0.024 (0.014**–**0.034)**	**0.017 (0.003**–**0.030)**	− 0.002 (− 0.017 to 0.015)	− 0.011 (− 0.026 to 0.005)
Relative Humidity (linear)	1.518 (− 0.185 to 3.328)	**2.135 (0.425**–**3.999)**	**2.443 (0.710**–**4.347)**	**2.117 (0.394**–**3.998)**
Relative Humidity (quadratic)	**− 1.896 (− 3.685 to − 0.024)**	− 0.533 (− 2.290 to 1.232)	− 0.617 (− 2.398 to 1.167)	− 0.245 (− 1.993 to 1.474)
Sex	**− 0.238 (− 0.394 to − 0.083)**	**− 0.264 (− 0.422 to − 0.108)**	**− 0.283 (− 0.438 to − 0.128)**	**− 0.285 (− 0.441 to − 0.131)**

During autumn migration, the two-way interaction of sex and age was included. Mean estimates and CrI are presented for each explanatory variable (transformed values; see methods). Effects (CrI do not include zero) are shown in bold. Reference category for age was first-years and for sex males

When comparing nights with favourable (n-rhum and/or favourable winds) and unfavourable (h-rhum and/or adverse winds) weather in each season, only the flight paths aligned with the nearest coastal destinations showed lower arrival fuel loads (autumn, Wangerooge—n-rhum: n = 115, estimate mean = 0.085, CrI = 0.075–0.095; h-rhum: n = 76, 0.086, CrI = 0.076–0.098; spring, St. Peter-Ording—n-rhum: n = 45, 0.056, CrI = 0.042–0.072; h-rhum: n = 10, 0.062, CrI = 0.046–0.082) for birds arriving after nights with adverse winds than after nights with favourable winds (autumn—n-rhum: n = 280, 0.109, CrI = 0.101–0.117; h-rhum: n = 448, 0.110, CrI = 0.103–0.117; spring—n-rhum: n = 111, estimate mean = 0.109, CrI = 0.101–0.117; h-rhum: n = 227, 0.110, CrI = 0.103–0.117; Additional file 1: Fig. S2, Additional file 1: chapter 6).

When comparing weather days for flight paths aligned with the other three coastal destinations in autumn, no differences were found. In spring, they showed weak (Amrum—n-rhum: n = 114, 0.095, CrI = 0.083–0.109; h-rhum: n = 252, 0.118, CrI = 0.108–0.129; Mandal—n-rhum: n = 101, 0.093, CrI = 0.080–0.109; h-rhum: n = 255, 0.112, CrI = 0.104–0.122) or strong effects (Blåvand—n-rhum: n = 134, 0.091, CrI = 0.079–0.103; h-rhum: n = 242, 0.112, CrI = 0.106–0.127) when comparing relative humidity conditions on nights with favourable winds. On the other hand, no differences were found between relative humidity conditions on nights with prevailing adverse winds, possibly due to the small annual sample sizes caught after such nights (n-rhum: 0–45 individuals; h-rhum: 10–24, even zero for Blåvand). This was due both to few nights with adverse winds (on 3–10 days) and to the fact that only few individuals were caught the following day (only 1–4 days with more than 5 individuals caught).

### Weather effects on percentage distribution and simulated successful flight rate

**Fig. 4:**
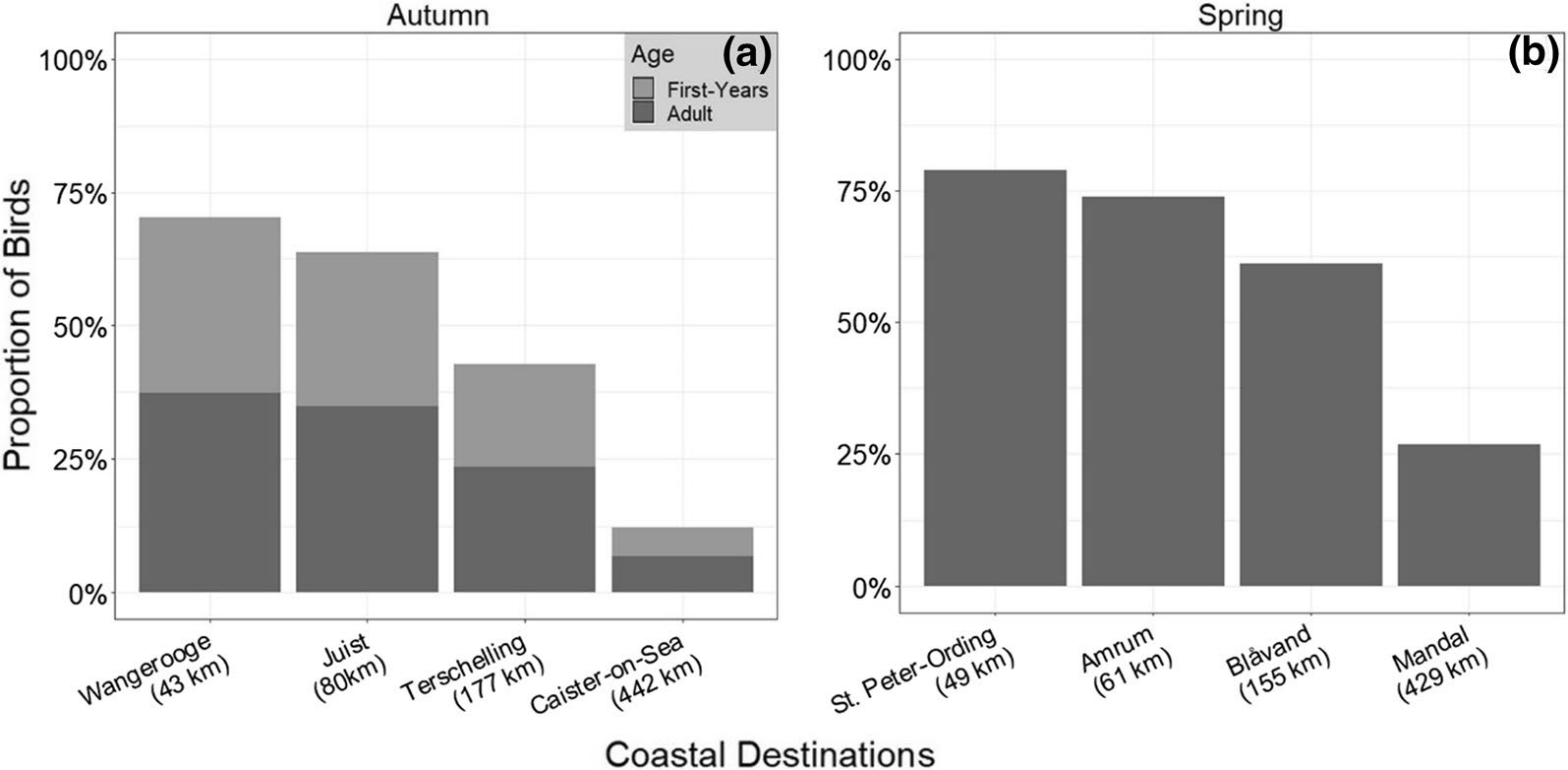
Proportion of blackbirds that would have been able to reach each coastal destination with different flight distance, respectively, during **a** autumn (n = 919) and **b** spring migration (n = 393), based on simulated trajectories with flow-assistance by real wind conditions. Age differentiation is only possible in autumn (first-years n = 461; adults n = 458)

Under the directional flow-assistance simulated for individual birds during the presumed migration night before stopping on Helgoland and their individual arrival fuel load, 70% of the blackbirds caught in autumn and 79% in spring would have successfully reached the nearest coastal destinations (Wangerooge and St. Peter-Ording; Fig. 4), while only 12% and 27% of the birds, respectively, would have reached the furthest coastal destinations (Caister-on-Sea and Mandal). Depending on the associated flow-assistance, all sex and age groups had lower rates of successful flights compared to still air condition by eleven to 14 percentage points in spring and eleven to 23 percentage points in autumn (Additional file 1: Table S5, Additional file 1: chapter 6).

During both seasons, the fuel loads of blackbirds stopping on Helgoland differed after nights with favourable and unfavourable weather conditions: considering the flight path directed towards the nearest coastal destination, Wangerooge and St. Peter-Ording, proportion of lean birds (≤ 5% arrival fuel load) stopping on Helgoland was higher after nights with adverse winds (autumn: 34%, spring: 64%; Additional file 1: Fig. S3a,c, Additional file 1: chapter 6) than when winds were favourable (autumn: 25%, spring: 23%; Additional file 1: Fig. S3b,d). Independent of wind conditions, most individuals with arrival fuel loads > 25% were caught after nights with h-rhum. Overall rate of successful flights, i.e. across all fuel load classes, was also in both season higher in blackbirds caught during nights with favourable winds (autumn: 75%, spring 92%) than in adverse winds (autumn: 54%, spring: 9%). This overall rates decreased with increasing flight distance of the other coastal destinations during autumn (Juist—favourable winds: 74%, adverse winds: 38%; Terschelling—53% and 15%; Caister-on-Sea—17% and 1%) and spring migration (Amrum: 77% and 19%; Blåvand: 61% and 12%; Mandal: 26% and 8%).

## Discussion

In this study, we were able to help decipher how physiological condition, wind and relative humidity force commen blackbirds to stop on a remote island. By directly measuring the individuals’ arrival fuel load using QMR, we were able to simulate the birds’ successful flight, assuming that they would not have stopped on Helgoland. This allowed us to separately consider two important aspects (energy limitation and weather as a flight obstruction) that influence different landing decisions in birds. As expected, we were able to show that, depending on the selected possible flight path, adverse winds tended to affect the lean birds with low energy resources, while poor visibility, i.e. high relative humidity, affected birds in general, regardless of whether the arrival fuel load was sufficient to allow onward flight. Furthermore, we could show that part of the variance found could be explained by minor secondary factors like age, sex and season, suggesting differentiated migratory strategies.

### Effect of arrival fuel load as energy limitation

In still air, over 90% of the birds stopping on Helgoland in both seasons carried sufficient fuel loads (≥ 1%) to continue their travel to the nearest selected coastal destinations (Fig. 2), and 36% would even reach the furthest coastal destination in the UK or Norway directly in a non-stop flight (sufficient fuel loads ≥ 13%). Therefore, although ¼ of the blackbirds arriving on Helgoland can be considered lean (≤ 5% arrival fuel load), most birds would not have needed to land on Helgoland as an emergency stopover for refuelling [10] due to limited energy reserves. Our results under still air support the statement of Dierschke and Bindrich [38] that fuel load alone does not have a strong influence on the birds’ decision to land on Helgoland, as the further sea crossing represents only a short hop. Thus, the questions to be discussed are why birds that are not constrained by insufficient energy reserves for a successful onward flight stop on Helgoland, and to what extent the weather influences this choice.

When interpretating such energy reserves, it should be noted that in our study, we equate fuel load with the birds’ fat amount. However, it is known that migrating birds can also store proteins, which are predominantly catabolised from muscles and digestive organs [72, 73], as an energy reserve for flight. Excluding proteins as fuel load may lead to an underestimation of the birds’ total fuel loads and maximum flight ranges calculated from them. Salewski et al. [74] showed that including muscle mass as fuel load resulted in a 35% higher mean potential flight range of four garden warblers in the desert. Therefore, a higher percentage of the blackbirds studied here may have been potentially able to reach coastal destinations than estimated. Yet, as garden warblers are long-distance migrants, simply adopting these assessments for the blackbirds in our study would almost certainly be incorrect, especially as proteins contribute only around 5% of the energy needed [75]. In general, the inclusion of protein as fuel load would only lead to a relative shift of our results without affecting the basic statement of our study.

### Effect of winds as flight obstruction

Similar to studies on departure decisions [18, 21, 23], we were able to highlight wind as an important driver of the decision to stop on Helgoland when analysing flight paths directed towards Wangerooge (autumn migration) as well as St. Peter-Ording and Amrum (spring migration). Here, TWC experienced the night before landing on Helgoland in both seasons correlated positively with the arrival fuel load of blackbirds caught the following day (Table 2, Fig. 3a,c). Thereby, our results indicate that especially lean birds are influenced by strong headwinds.

Interestingly, this was not the case for the other coastal destinations. Since we do not exactly know where each individual bird actually comes from and coastal destinations were selected in this study on the basis of ringing data, we cannot clearly explain why wind influences were only found for the flight paths that were aligned with the less distant coastal destinations (Fig. 1). It is conceivable that the majority of our observed blackbirds stopping on Helgoland actually flew on these flight paths around the time of the weather measurements (00:00 UTC) before landing. This would only be possible if a) the birds actually departed from a starting point that was on the opposite side of the theoretical flight path (autumn: Norway, spring: UK or German-Dutch border) or/and b) the birds travelled along the coast and drifted into our flight path at some point before Helgoland. In both cases, fuel loads of some birds will have been dramatically depleted by strong headwinds [27–29]. (Nocturnal) birds in such a situation should try to reorient themselves parallel to the coastline [15, 40] towards the nearest coastal destination. Therefore, birds driven by adverse winds to a point in front of Helgoland might actually have oriented themselves towards Wangerooge and St. Peter-Ording (possibly also Amrum) before deciding to stop over at Helgoland, as these are the quickest to reach with only 43 to 61 km flight distance (Fig. 1) and have the highest success flight rate. This would be in line with results from Eikenaar et al. [48], who observed a departure direction of 181° of radio-tagged blackbirds on Helgoland during autumn migration (Wangerooge: 178°; Additional file 1: Table S3, Additional file 1: chapter 4). Radar observations on Helgoland during autumn migration also revealed that, besides the main south-westerly direction, a considerable proportion of nocturnal migrants fly in a southerly direction [76]. Interestingly, Hüppop and Hilgerloh [12] calculated a different autumn migration direction of 235° from recaptures within one month after ringing on Helgoland. However, birds can fly detours and adjust flight directions several times during such a long-time interval. Thus, this mean total flight direction can be divided into two (or more) travel intervals: blackbirds (1) departing towards the nearest coastal destination, which would be consistent with Eikenaar et al. [48], part of the radar observation [77] and our results; (2) adjusting their flight direction towards the next stopover or final destination after reaching “safe” terrain. Further studies tracking blackbirds caught in their breeding areas are needed to identify preferred flight paths and from this the reasons why blackbirds fly over Helgoland. These studies should thereby include possible age-differences, as especially first-year birds might tend to choose shorter over-water crossings, as lack of ability and experience pose a greater risk than in adult birds [78]. Another aspect to consider is that we do not know the individual origins of our blackbirds. Different populations stopping on Helgoland may have different flight paths over the sea. Therefore, missing relationships between TWC and some coastal destinations could be due to the fact that we assumed the same migratory pattern for all individuals in our study, which is probably not the case.

Nevertheless, our results in general indicate that lean birds may not have the energy reserves to compete against strong headwinds and compensate for drift by strong crosswinds, as strong head- and crosswinds make orientation difficult, reduce the birds’ groundspeed and thus increase fuel consumption per unit distance [22, 27]. Therefore, they are likely blown offshore towards Helgoland if winds were too strong [22, 79]. In addition to these already lean birds, it is also conceivable that previously better conditioned blackbirds suffered large energy losses due to increased energy consumption under strong head- and crosswinds and thus perceived Helgoland as an unplanned stopover to refuel.

The effect of such adverse winds on the probable mortality risk of terrestrial sea-crossing migrants is implied in our study by the reduced proportion of successful simulated flights in prevailing winds during the night (Fig. 4): while only 9% of all blackbirds captured on Helgoland did not have sufficient fuel loads to allow them to safely travel on in still air (see previous chapter), wind conditions would have prevented 30% of birds during autumn migration and 21% during spring migration from successfully reaching the nearest coastal destination, with the situation further deteriorating with increasing flight distance (other coastal destinations). The lower success rate during autumn migration results from the prevailing westerly and south-westerly winds typical of Central Europe during this season, which are experienced as head- or crosswinds [34, 80] by blackbirds migrating mainly WSW from Scandinavia [30, 62, 81]. In our study, such winds inhibited successful flights on 24 to 65% of the 123 autumn nights observed. Spring migration showed a more supporting pattern with 57 to 92% of the 83 observed nights allowing successful flights. This reflects the more profitable and beneficial spring winds when blackbirds cross the North Sea with the main direction of avian migration in NE [30, 62, 81]. As wind conditions in spring of our observed years were less favourable than typically observed for Helgoland during this season [30], successful flights might be, on average over the years, possible on more days and thus also for a larger proportion of birds.

Erni et al. [39] hypothesised that a flow-assistance ≤ -5 m/s can be considered stressful and a limiting threshold for migrating birds. Although TWC does not account for crosswinds, it is striking that birds caught on days following nights with such adverse winds (≤ -5 m/s) carried on average lower arrival fuel loads than when winds were favourable (Additional file 1: Fig. S2, Additional file 1: chapter 6), which also resulted in a higher proportion of lean birds (Additional file 1: Fig. S3), when considering TWC towards the nearest coastal destinations. Furthermore, the simulated possibility of successfully continuing on such nights and reaching at least the nearest coastal destination was greatly reduced, especially for lean birds. Therefore, our results support the hypothesis of Erni et al. [39], as they indicate a higher probable mortality risk for blackbirds flying under adverse winds.

Our findings on the influence of TWC complement those of previous studies: while for blackbirds and other thrushes the decision to depart (and probably also to continue or fly over) is favoured for an oversea crossing in weak and/or tailwind conditions [12, 21, 23], landing on an island en route should be favoured in strong headwinds—especially when energy reserves are low and probable mortality risk thus is high, as wind is considered the greatest determinant of annual apparent survival [45]. However, Dierschke and Bindrich [38] caught the heaviest birds on Helgoland in unfavourable winds. One reason for the differences with our study could be that this tendency towards heavier birds was based on big fall days (i.e. days with > 200 individuals caught). As these events were combined with high overcast conditions, this could be the actual driver behind this effect. Understanding the influence of relative humidity, being closely related to fog or (low-lying) cloud cover and precipitation, in addition to wind conditions is thus important.

### Additional effect of relative humidity as flight obstruction

In our study, all birds were affected by higher levels of relative humidity, regardless of the individuals’ arrival fuel load (Table 2, Fig. 3b,d). Furthermore, our results indicate that most blackbirds carrying largest fuel loads were found after nights with h-rhum (Additional file 1: Figs. S2, S3, Additional file 1: chapter 6), from which the majority would have been able to successfully travel on.

Interestingly, effects of relative humidity were often found for flight paths oriented towards coastal destinations that did not reveal effects of TWC (Table 2, Fig. 3). This suggests that the effect of relative humidity can be partially overridden by the effect of TWC. Here, the two weather parameters lead to effects on different sides of the arrival fuel load: while adverse winds affect the lean birds, most birds, including the fat ones, are additionally influenced by h-rhum. Thus, if TWC is the major influence [82], it may override “minor” effects of relative humidity.

Nevertheless, our results show that well-conditioned individuals (i.e. large fuel loads) stopped on Helgoland not due to energy limitation or adverse winds, but rather due to high relative humidity. As h-rhum is strongly associated with heavy cloud cover, fog, drizzle and precipitation [12, 70, 71], this condition coincides with poor visibility, which is generally detrimental for orientation of migrating birds [83], and “forces” even fat birds to land on Helgoland. Brust et al. [23] also found that 65% of the birds flew on days with relative humidity below 80%. As fewer birds appear to fly under such conditions [15, 84], birds may use Helgoland as an emergency stopover site regardless of their physiological condition, as it seems wiser to take off again when visibility is restored by clear skies [23]. Other related aspects such as increased flight costs in very humid air, which could affect the birds’ flight capability and thermoregulation, can be additionally weighed for the birds’ landing decision [7, 39].

### Effects of season, age and sex

Although we were able to disentangle the effects of weather and arrival fuel load on the decision of blackbirds to stop, high variation remained. Therefore, sex and age classes need to be considered in a seasonal context, as they may have different migratory strategies and therefore may cope differently with different environmental situations [85].

Assuming still air, seasonal-dependent differences in arrival fuel loads were observed between sex and age groups (Table 1): adults carried larger fuel loads than first-years because they were possibly better prepared, most likely due to more experience [42, 43]. This would have enabled a higher proportion of the adults stopping on Helgoland to successfully reach a coastal destination without refuelling, indicating a reduction in the “mortality risk” compared to first-years. First-years, on the other hand, may choose shorter over-water crossings with less navigational risk [78] and less energy reserves required. It should be kept in mind that birds with very large fuel loads usually do not stop on Helgoland, as shown by the high fat score levels of blackbirds that crashed with an offshore platform near the island [86]; therefore, our data represent a possible “capture bias”.

While no sex-specific differences were evident during autumn migration, males carried larger arrival fuel loads than females during spring migration. Seasonal differences often represent different migratory strategies within a species, as in spring the timing of breeding and thus of arrival at the breeding areas is relevant for individual fitness due to carry-over effects between migration and breeding [87, 88]: a later arrival at the breeding grounds leads to later breeding and ultimately to lower breeding success than earlier breeding conspecifics [45]. Therefore, larger fuel loads allow males in particular, which are under greater evolutionary pressure, to reach breeding areas earlier than females and establish themselves in prime territories [3, 44, 89, 90].

Once wind was included to simulate trajectories, the rates of successful flights of all blackbird groups were negatively affected in both seasons. Sex and age differences in still air (see above) persisted, so that males had higher simulated success flight rates than females and adults had higher rates than first-years. We would have expected first-years either to show larger variance in simulated success flight rates than adults when wind effects were included, as they fly for the first time and “poor quality individuals” were not yet “weeded out” by natural selection, or to show larger decreases, since they are assumed to reach their final destination purely through endogenous programmes, including decisions about timing, flight direction and duration, whereas adults modify this programme by additionally developing a map through the experience of previous migration flight [32, 91, 92]. Therefore, unlike adults, first-years should lack the experience to correctly judge wind conditions as (not) supportive and thus select certain more supportive winds and adjust their wind selection criteria [34, 93]. This hypothesis is supported by previous studies showing that first-years are less wind selective than adults at departure and have considerably less efficient migratory flights [48, 94, 95]. However, we found no evidence of this in our study.

In contrast to within-seasonal differences found in term of age and sex, between-seasonal differences were not present. This is not surprising considering that the birds’ arrival fuel load observed on Helgoland can vary due to many parameters, e.g. flight route and wind conditions [27–29] as well as departure fuel load at the beginning of the oversea crossing. Further on, as the blackbirds are likely forced to land at least in part due to energy limitations or flight obstructions, possible seasonal effects are likely overridden by these main effects.

## Conclusion

Our results suggest that common blackbirds not only use Helgoland as an emergency stopover site for refuelling [10], but also at least partly as the first suitable stopover when weather conditions become unfavourable. However, “unfavourable” seems to depend on the birds’ available fuel load: while headwinds, which increases fuel consumption of birds [27], resulted in more lean birds stopping on Helgoland, unable to continue flying in such winds, blackbirds in general were rather affected by high relative humidity, as a proxy for poor visibility compromising orientation, regardless of fuel load, i.e. including birds carrying large amounts of fat. Our results provide a new perspective on previously statements that fuel load has no influence on the birds’ decision to land on a remote island where further sea crossing is only a seemingly short ecological barrier [38]. This may be the case when considering purely still air conditions. But to successfully reach a destination, not only the fuel load but also the corresponding real wind conditions have to be considered. In this case, the fuel load has an influence on how the birds cope with certain wind conditions.

Of course, the blackbird is only one of many passerine species stopping over on Helgoland, differing in their breeding and wintering areas, with some of them having to cross the Mediterranean Sea and/or the Sahara Desert as major ecological barriers. Here, other bird species might show different migratory strategies in dealing with unfavourable weather. Therefore, we encourage repeating this study in a multi-species approach, comparing different types like short/medium-distance and trans-Saharan migrants or diurnal versus nocturnal migrants. Future studies could also focus on what is the physiological condition of (black)birds that do not stop on a remote island like Helgoland but fly on, and whether their migratory strategy differs from that of landing birds. We would expect these birds to show partly complementary results to our findings, e.g. larger fuel loads [86] as they have to reach the coast from then on due to a lack of further stopping opportunities.

In summary, understanding the local weather and individuals’ physiological conditions that lead to an interruption of migration, e.g. on an island like Helgoland, will help to understand the different observed migratory strategies and explain further migratory questions such as stopover duration, departure and total migration timing.

## Supplementary Information

**Additional file 1: Chapter 1**. Method flowchart. **Fig. 1**. Supporting flowchart for overview of the methods. **Chapter 2**. Information on EchoMRI™. Details on functionality, software settings and validation. **Chapter 3**. Overview of weather parameters. Details on (initially) used weather parameters. **Table 1**. Variation in weather parameters for each investigated pressure level. **Table 2**. Variation in analysed altitude-dependent weather conditions. **Chapter 4**. Details to coastal destinations. **Table 3**. Coordinates, flight distances and flight direction for selected coastal destinations. **Chapter 5**. Overview of average fuel-dependent flight range. Range of possible flight range and the birds’ fuel loads depending on fat score levels. **Table 4**. Fat score levels, absolute and (relative) arrival fuel load and potential flight range. **Chapter 6**. Excluded parameters and additional results. Details in initially included parameters. **Table 5**. Successful flight rates. **Fig. 2**. Arrival fuel loads following nights with un- and favourable weather. **Fig. 3**. Distribution of arrival fuel load depending on weather conditions.

## Data Availability

The dataset analysed during the current study are available on reasonable request.

## Abbreviations

QMR: Quantitative magnetic resonance technology used by the EchoMRI™
TWC: Tailwind components
CrI: Credible intervals
LM: Linear models
GLM: Generalised linear models
h-rhum: “High” relative humidity ≥ 80%
n-rhum: “Normal” relative humidity < 80

## References

[1] Moore F, Kerlinger P (1987). Stopover and fat deposition by North American Wood-Warblers (*Parulinae*) following spring migration over the Gulf of Mexico. Oecologia.

[2] Hedenström A (2010). Extreme endurance migration: what is the limit to non-stop flight?. PLoS Biol.

[3] Alerstam T (2011). Extreme endurance migration: what is the limit to non-stop flight?. PLoS Biol.

[4] Ward MP, Benson TJ, Deppe J, Zenzal TJ, Diehl RH, Celis-Murillo A (2018). Estimating apparent survival of songbirds crossing the Gulf of Mexico during autumn migration. Proc R Soc B Biol Sci.

[5] Bradarić M, Bouten W, Fijn RC, Krijgsveld KL, Shamoun-Baranes J (2020). Winds at departure shape seasonal patterns of nocturnal bird migration over the North Sea. J Avian Biol.

[6] Bulte M, McLaren JD, Bairlein F, Bouten W, Schmaljohann H, Shamoun-Baranes J (2014). Can wheatears weather the Atlantic? Modeling nonstop trans-Atlantic flights of a small migratory songbird. Auk.

[7] Deppe JL, Ward MP, Bolus RT, Diehl RH, Celis-Murillo A, Zenzal TJ (2015). Fat, weather, and date affect migratory songbirds’ departure decisions, routes, and time it takes to cross the Gulf of Mexico. PNASUSA.

[8] Newton I (2007). Weather-related mass-mortality events in migrants. Ibis.

[9] La Sorte FA, Fink D, Hochachka WM, Kelling S (2016). Convergence of broad-scale migration strategies in terrestrial birds. Proc R Soc B.

[10] Hüppop O, Hüppop K (2011). Bird migration on Helgoland: the yield form 100 years of research. J Ornithol.

[11] Gätke H (1895). Heligoland as an ornithological observatory: The result of fifty years.

[12] Hüppop O, Hilgerloh G (2012). Flight call rates of migrating thrushes: effects of wind conditions, humidity and time of day at an illuminated offshore platform. J Avian Biol.

[13] Alerstam T (2001). Detours in bird migration. J Theor Biol.

[14] Gómez C, Bayly NJ, Norris DR, Mackenzie SA, Rosenberg KV, Taylor PD (2017). Fuel loads acquired at a stopover site influence the pace of intercontinental migration in a boreal songbird. Sci Rep.

[15] Richardson WJ (1978). Timing and amount of bird migration in relation to weather: a review. Oikos.

[16] Schaub M, Liechti F, Jenni L (2004). Departure of migrating European robins, Erithacus rubecula, from a stopover site in relation to wind and rain. Anim Behav.

[17] Haest B, Hüppop O, Bairlein F (2018). The influence of weather on avian spring migration phenology: what, where and when?. Glob Change Biol.

[18] Haest B, Hüppop O, van de Pol M, Bairlein F (2019). Autumn bird migration phenology: a potpourri of wind, precipitation and temperature effects. Glob Change Biol.

[19] Schmaljohann H, Korner-Nievergelt F, Naef-Daenzer B, Nagel R, Maggini I, Bulte M, Bairlein F (2013). Stopover optimization in a long-distance migrant: the role of fuel load and nocturnal take-off time in Alaskan Northern Wheatears (*Oeananthe oenanthe*). Front Zool.

[20] Sjöberg S, Alerstam T, Åkesson S, Schulz A, Weidauer A, Coppack T, Muheim R (2015). Weather and fuel reserves determine departure and flight decisions in passerines migrating across the Baltic Sea. Anim Behav.

[21] Packmor F, Klinner T, Woodworth BK, Eikenaar C, Schmaljohann H (2020). Stopover departure decisions in songbirds: do long-distance migrants depart earlier and more independently of weather conditions than medium-distance migrants?. Mov Ecol.

[22] Alerstam T (1976). Nocturnal migration of thrushes (*Turdus* spp.) in Southern Sweden. Oikos.

[23] Brust V, Michalik B, Hüppop O (2019). To cross or not to cross—thrushes at the German North Sea coast adapt flight and routing to wind conditions in autumn. Mov Ecol.

[24] Roques S, Henry P-Y, Guyot G, Bargain B, Cam E, Pradel R (2020). When to depart from a stopover site? Time-since-arrival matters more than weather conditions. bioRxiv.

[25] Liechti F (1995). Modelling optimal heading and airspeed of migrating birds in relation to energy expenditure and wind influence. J Avian Biol.

[26] Weber TP, Hedenström A (2000). Optimal stopover decisions under wind influence: the effects of correlated winds. J Theor.

[27] Bairlein F (2008). The mysteries of bird migration—still much be learnt. British Birds.

[28] Liechti F, Klaassen M, Bruderer B (2000). Predicting migratory flight altitudes by physiological migration models. Auk.

[29] Schmaljohann H, Liechti F, Bruderer B (2009). Trans-Sahara migrants select flight altitudes to minimize energy costs rather than water loss. Behav Ecol Sociobiol.

[30] Dierschke J, Dierschke V, Hüppop K, Hüppop O, Jachmann KF (2011). Die Vogelwelt der Insel Helgoland.

[31] Moore FR, Kerlinger P, Simons TR (1990). Stopover on a Gulf coast barrier island by spring trans-Gulf migrants. Wilson Bull.

[32] Berthold P (2001). Bird migration: a general survey.

[33] Lack D (1963). Migration across the southern North Sea studied by radar Part 5. Movements in August, winter and spring, and conclusion. Ibis.

[34] Kemp MU, Shamoun-Baranes J, van Gasteren H, Bouten W, van Loon EE (2010). Can wind help explain seasonal differences in avian migration speed?. J Avian Biol.

[35] Kemp MU, Shamoun-Baranes J, Dokter AM, van Loon E, Bouten W (2013). The influence of weather on the flight altitude of nocturnal migrants in mid-latitudes. Ibis.

[36] Manola I, Bradarić M, Groenland R, Fijn R, Bouten W, Shamoun-Baranes J (2020). Associations of synoptic weather conditions with nocturnal bird migration over the North Sea. Front Ecol Evol.

[37] Jellmann J, Vauk G (1978). Untersuchungen zum Verlauf des Frühjahrszuges über der Deutschen Bucht nach Radarstudien und Fang- und Beobachtungsergebnissen auf Helgoland. J Ornithol.

[38] Dierschke V, Bindrich F (2001). Body condition of migrant passerines crossing a small ecological barrier. Vogelwarte.

[39] Erni B, Liechti F, Underhill LG, Bruderer B (2002). Wind and rain govern the intensity of nocturnal bird migration in central Europe—a log-linear regression analysis. Ardea.

[40] Richardson WJ, Gwinner E (1990). Timing of bird migration in relation to weather: updated review. Bird migration.

[41] Fox J, Weisberg S (2019). An R companion to applied regression.

[42] Ellegren H (1991). Stopover ecology of autumn migrating Bluethroats *Luscinia* s. svecica in relation to age and sex. Ornis Scand.

[43] Ellegren H (1993). Speed of migration and migratory flight lengths of passerine birds ringed during autumn migration in Sweden. Ornis Scand.

[44] Kokko H, Gunnarsson TG, Morrell LJ, Gill JA (2006). Why do female migratory birds arrive lather than males?. J Animal Ecol.

[45] Drake A, Rock CA, Quinlan SP, Martin M, Green DJ (2014). Wind speed during migration influences the survival, timing of breeding, and productivity of a neotropical migrant *Setophaga petechia*. PLoS ONE.

[46] Hüppop K, Hüppop O (2009). Atlas zur Vogelberingung auf Helgoland Teil 5: Ringfunde von 1909 bis 2008. Vogelwarte.

[47] Raiss R (1979). Resting behaviour as an indicator for different migrational strategies in three species of European thrushes (*Turdus* sp). Abhandlungen aus dem Gebiet der Vogelkunde.

[48] Eikenaar C, Ballstaedt E, Hessler S, Klinner T, Mueller F, Schmaljohann H (2018). Cues, corticosterone and departure decisions in a partial migrant. Gen Comp Endocrinol.

[49] Dierschke V (2003). Predation hazard during migratory stopover: are light or heavy birds under risk?. J Avian Biol.

[50] Chernetsov N, Mukhin A (2006). Spatial behavior of European Robins during migratory stopovers: a telemetry study. Wilson J Ornithol.

[51] Bulyuk VN, Tsvey A (2013). Regulation of stopover duration in the European Robin *Erithacus rubecula*. J Ornithol.

[52] Svensson L (1992). Identification guide to European Passerines.

[53] EchoMRI. EchoMRI™—Corporation Pte Ltd., Body Composition Analysis. 2016. http://www.echomri.com. Accessed 8 July 2020.

[54] Guglielmo CG, McGuire LP, Gerson AR, Seewagen CL (2011). Simple, rapid, and non-invasive measurement of fat, lean, and total water masses of live birds using quantitative magnetic resonance. J Ornithol.

[55] Kelsey NA, Bairlein F (2019). Migratory body mass increase in Northern Wheatears (*Oenanthe oenanthe*) is the accumulation of fat as proven by quantitative magnetic resonance. J Ornithol.

[56] Kelsey NA, Schmaljohann H, Bairlein F (2019). A handy way to estimate lean body mass and fuel load from wing length: a quantitative approach using magnetic resonance data. Ringing Migr.

[57] Kemp MU, Emiel van Loon E, Shamoun-Baranes J, Bouten W (2012). RNCEP: global weather and climate data at your fingertips. Methods Ecol Evol.

[58] Kalnay E, Kanamitsu M, Kistler R, Collins W, Deaven D, Gandin L (1996). The NCEP/NCAR 40-year reanalysis project. Bull Am Meteorl Soc.

[59] Bruderer B, Peter D, Korner-Nievergelt F (2018). Vertical distribution of bird mgiration between the Baltic Sea and the Sahara. J Ornithol.

[60] Bruderer B, Boldt A (2001). Flight characteristics of birds: I radar measurements of speeds. Ibis.

[61] Erni B, Liechti F, Bruderer B (2005). The role of wind in passerine autumn migration between Europe and Africa. Behav Ecol.

[62] Bairlein F, Dierschke J, Dierschke V, Salewski V, Geiter O, Hüppop K (2014). Atlas des Vogelzuges. Ringfunde deutscher Brut- und Gastvögel.

[63] Bakken V, Runde O, Tjørve E (2006). Norsk ringmerkingsatlas (Norwegian bird ringing atlas).

[64] Fransson T, Hall-Karlsson S (2008). Swedish bird ringing atlas.

[65] Kemp MU, Shamoun-Baranes J, van Loon EE, Bouten W (2012). Quantifying flow-assistance and implications for movement research. J Theor.

[66] Delingat J, Bairlein F, Hedenström A (2008). Obligatory barrier crossing and adaptive fuel management in migratory birds: the case of the Atlantic crossing in Northern Wheatears (*Oenanthe Oenanthe*). Behav Ecol Sociobiol.

[67] R Development Core Team. R: a language and environment for statistical computing. Vienna, Austria: R Foundation for Statistical Computing, version 4.0.2, 2020. http://www.R-project.org. Accessed 08. August 2020.

[68] Korner-Nievergelt F, Roth T, von Felten S, Guélat J, Almasi B, Korner-Nievergelt P (2015). Bayesian data analysis in ecology using linear models with R, BUGS, and Stan.

[69] Zuur AF, Ieno EN, Elphick CS (2010). A protocol for data exploration to avoid common statistical problems. Methods Ecol Evol.

[70] Inoue T, Kamahori H (2001). Statistical relationship between ISCCP cloud type and vertical relative humidity profile. J Meteorol.

[71] Teixeira J (2001). Cloud fraction and relative humidity in a prognostic cloud fraction scheme. Mon Weather Rev.

[72] Hume ID, Biebach H (1996). Digestive tract function in the long-distance migratory Garden Warbler *Sylvia borin*. J Comp Physiol B.

[73] Piersma T, Gill RE (1998). Guts don’t fly: small digestive organs in obese Bar-tailed Godwits. Auk.

[74] Salewski V, Herremans M, Liechti F (2010). Migrating passerines can lose mor body mass reversibly than previously thought. Ringing Migr.

[75] Jenni L, Jenni-Eiermann S (1998). Fuel supply and metabolic constraints in migrating birds. J Avian Biol.

[76] Hüppop K, Hüppop O (2004). Atlas zur Vogelberingung auf Helgoland Teil 2: Phänologie im Fanggarten von 1961 bis 2000. Vogelwarte.

[77] Hüppop O, Dierschke J, Wendeln H (2005). Zugvögel und Offshore-Windkraftanlagen: Konflikte und Lösungen. Ber Vogelschutz.

[78] Crysler ZJ, Ronconi RA, Taylor PD (2016). Differential fall migratory routes of adult and juvenile Ipswich Sparrows (*Passerculus sandwichensis* princeps). Mov Ecol.

[79] Scholander SI (1995). Land birds over the western North Atlantic. Auk.

[80] Fitzroy R (2012). The weather book: a manual of practical meteorology.

[81] Main IG (2002). Seasonal movements of Fennoscandian Blackbirds *Turdus merula*. Ring Migrat.

[82] Van Belle J, Shamoun-Baranes J, Van Loon E, Bouten W (2007). An operational model predicting autumn bird migration intensities for flight safety. J Appl Ecol.

[83] Åkesson S, Bäckman J (1999). Orientation in Pied Flycatchers: the relative importance of magnetic and visual information at dusk. Anim Behav.

[84] Baumgartner M (1997). Wetterabhängigkeit des nächtlichen Vogelzuges im Herbst über Süddeutschland (Inauguraldissertation).

[85] Shamoun-Baranes J, Liechti F, Vansteelant WM (2017). Atmospheric conditions create freeways, detours and tailbacks for migrating birds. J Comp Physiol A.

[86] Hüppop O, Hüppop K, Dierschke J, Hill R (2016). Bird collisions at an offshore platform in the North Sea. Bird Study.

[87] Fransson T (1995). Timing and speed of migration in North and West European populations of *Sylvia* warblers. J Avian Biol.

[88] Drent RH (2006). The timing of birds’ breeding seasons: the Perrins hypothesis revisited especially for migrants. Ardea.

[89] Francis CM, Cooke F (1986). Differential timing of spring migration in Wood Warblers (*Parulinae*). Auk.

[90] Spina F, Massi A, Montemaggiori A (1994). Back from Africa: Who’s running ahead? Aspects of differential migration of sex and age classes in Palearctic-African spring migrants. Ostrich.

[91] Wiltschko R, Wiltschko W. Avian navigation: from historical to modern concepts. In: Lucas JR, Simmons LW, editors. Essays in animal behaviour—celebrating 50 years of animal behaviour; 2003. p. 257–272.

[92] Liechti F (2006). Birds: blowin’ by the wind?. J Ornithol.

[93] Eikenaar C, Richardson DS, Brouwer L, Komdeur J (2008). Sex biased natal dispersal in a closed, saturated population of Seychelles Warblers *Acrocephalus sechellensis*. J Avian Biol.

[94] Mitchell GW, Woodworth BK, Taylor PD, Norris DR (2015). Automated telemetry reveals age specific differences in flight duration and speed are driven by wind conditions in a migratory songbird. Mov Ecol.

[95] Mitchell GW, Newman AEM, Wikelski M, Norris DR (2012). Timing of breeding carries over to influence migratory departure in a songbird: an automated radiotracking study. J Animal Ecol.

