# Supplementary material for: Days to visit an offshore island: effect of weather conditions on arrival fuel load and potential flight range for common blackbirds Turdus merula migrating over the North Sea
Source: Mov Ecol. 2021 Oct 21;9:53. doi: 10.1186/s40462-021-00290-6 (PMC8529821; doi:10.1186/s40462-021-00290-6)
Supplement: Supplementary file 1 — Additional file 1: Chapter 1. Method flowchart. Fig. 1. Supporting flowchart for overview of the methods. Chapter 2. Information on EchoMRI™. Details on functionality, software settings and validation. Chapter 3. Overview of weather parameters. Details on (initially) used weather parameters. Table 1. Variation in weather parameters for each investigated pressure level. Table 2. Variation in analysed altitude-dependent weather conditions. Chapter 4. Details to coastal destinations. Table 3. Coordinates, flight distances and flight direction for selected coastal destinations. Chapter 5. Overview of average fuel-dependent flight range. Range of possible flight range and the birds’ fuel loads depending on fat score levels. Table 4. Fat score levels, absolute and (relative) arrival fuel load and potential flight range. Chapter 6. Excluded parameters and additional results. Details in initially included parameters. Table 5. Successful flight rates. Fig. 2. Arrival fuel loads following nights with un- and favourable weather. Fig. 3. Distribution of arrival fuel load depending on weather conditions. [file 40462_2021_290_MOESM1_ESM.pdf]

Article type: Research

Additional file: Days to visit an offshore island: Effect of weather conditions on arrival fuel load and potential flight range for common blackbirds *Turdus merula* migrating over the North Sea

Chapter 1: Method flowchart

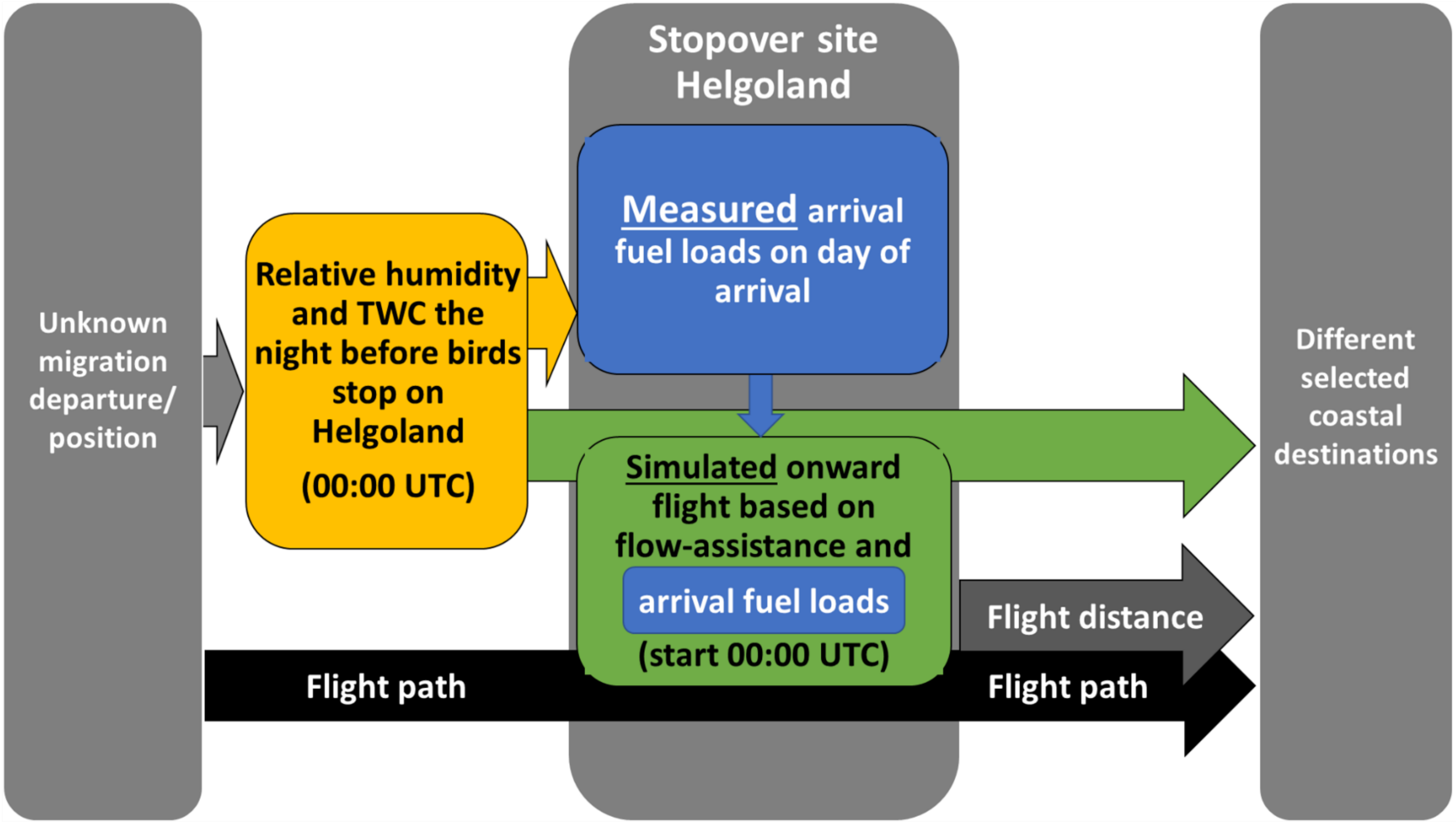

Supplementary Fig. 1: Flow chart for overview for better understanding of the method section. With the arrows and flow chart parts we outline the respective temporal and spatial course of our study: 1) take-off of the blackbirds at an unknown position ahead of Helgoland, 2) approach to Helgoland the night before landing (yellow) oriented towards the selected potential flight paths (black), 3) actual stopover on Helgoland and capture the following day (blue) or 4) simulation of a continuation of the flight over Helgoland (including arrival fuel load as energy limitation) oriented towards the selected potential flight paths (including flight distance, grey) towards 5) the selected coastal destinations. Each of the coloured boxes includes point in time and the parameters measured at that point.

Chapter 2: Information on EchoMRI™

For this scanning procedure, we used an appropriately sized ventilated holding tube and the associated 'BIRD' setting of the EchoMRI™ software (accumulation setup: three), cf. Kelsey et al. (2019). Repeatability of the absolute fuel load within the three scans was high (= 0.99).

Required is a weekly calibration of the QMR device using the software 'system test' and the manufacturer's canola oil standard as well as a daily calibration prior to the start of actual scanning. See Guglielmo et al. (2011), Seewagen & Guglielmo (2011) and Kelsey & Bairlein (2019) for more details on the correct use of the EchoMRI™.

Further details on the validation and physics of the QMR technique can be traced in Taicher et al. (2003) and Tinsley et al. (2004).

Guglielmo CG, McGuire LP, Gerson AR, Seewagen CL. Simple, rapid, and non-invasive measurement of fat, lean, and total water masses of live birds using quantitative magnetic resonance. J Ornithol. 2011;152:75-85.

Kelsey NA, Bairlein F. Migratory body mass increase in Northern Wheatears (*Oenanthe oenanthe*) is the accumulation of fat as proven by quantitative magnetic resonance. J Ornithol. 2019;160:389-397.

Kelsey NA, Schmaljohann H, Bairlein F. A handy way to estimate lean body mass and fuel load from wing length: a quantitative approach using magnetic resonance data. Ringing Migr. 2019;34:8-24.

Seewagen CL, Guglielmo CG. Quantitative magnetic resonance analysis and a morphometric predictive model reveal lean body mass changes in migrating Nearctic–Neotropical passerines. J Comp Physiol B. 2011;181:413-421.

Taicher GZ, Tinsley FC, Reiderman A, Heiman ML. Quantitative magnetic resonance (QMR) method for bone and whole-body-composition analysis. **Anal. Bioanal. Chem**. 2003;377:990–1002.

Tinsley FC, Taicher GZ, Heiman ML. Evaluation of a quantitative magnetic resonance method for mouse whole body composition analysis. Obesity Research. 2004;12:150-160.

Chapter 3: Overview of weather parameters

Temperature ("air"; K) was not included in the analyses because birds can fly at air temperatures below -15 °C (Bruderer 1971) and above 40 °C (Whitfield et al. 2015), while air temperatures ranged from -13.95 °C (including only two nights with below -10.00 °C) to 19.45 °C during our observation periods (supplementary Table 1). Metabolic rates of flying birds are 5-10 times higher than resting rates, substituting for regular thermogenesis (Dawson & Connor 1996). Therefore, dramatically increased energetic costs of thermoregulation as well as possible ice accumulation on the plumage (Elkins 2004), which could lead to a "forced" landing of the birds, are unlikely. The same applies to possible hyperthermia with heat stress effects (Whitfield et al. 2015), as hyperthermia contributes little to the cost of thermoregulation in birds up to around 100 g (Weathers 1981).

Furthermore, precipitation, cloud cover and cloud altitude were not used because these are not directly assimilated but derived entirely from the predictions of the NCEP reanalysis models and are therefore less reliable (Kalnay et al. 1996; Kistler et al. 2001; Hüppop & Hilgerloh 2012).

Bruderer B. Radarbeobacthungen über den Frühlingszug im schweizerischen Mittelland (Ein Beitrag zum Problem der Witterungsabhängigikeit des Vogelzuges). Ornithol Beob. 1971;68:89-158.

Dawson WR, O'Connor TP. Energetic features of avian thermoregulatory responses. In: Carey C, editor. Avian energetics and nutritional ecology. Weinheim, Germany: Chapman & Hall GmbH; 1996. p. 85-124.

Elkins N. Weather and bird behaviour. London, Oxford: Bloomsbury Publishing; 2010.

Hüppop O, Hilgerloh G. Flight call rates of migrating thrushes: effects of wind conditions, humidity and time of day at an illuminated offshore platform. J Avian Biol. 2012;43:85-90.

Kalnay E, Kanamitsu M, Kistler R, Collins W, Deaven D, Gandin L, et al. The NCEP/NCAR 40-year reanalysis project. Bull Amer Meteor Soc. 1996;77:437-470.

Kistler R, Kalnay E, Collins W, Saha S, White G, Wollen J, et al. The NCEP-NCAR 50-year reanalysis. Monthly means CD-ROM and documentation. Bull Am Meteorl Soc. 2001;82:247-267.

Weathers WW (1981) Physiological thermoregulation in heat-stressed birds: consequences of body size. Physiological Zoology 54, 345-361.

Whitfield MC, Smit B, McKechnie AE, Wolf BO. Avian thermoregulation in the heat: scaling of heat tolerance and evaporative cooling capacity in three southern African arid-zone passerines. J Exp Biol. 2015;218:1705-1714.

Supplementary Table 1. Variation in weather parameters measured each night before bird capture for the three investigated pressure levels (1000, 925 and 850 hPa) per season. Given are the mean ± standard deviation as well as the range (in brackets) of the variation in each variable. Tailwind components are calculated using the direction of the flight paths aligned with the selected coastal destinations (Fig. 1; see methods).

					**Tailwind components (m/s)**			
**Autumn**	**Temperature (°C)**	**Relative Humidity (%)**	**U-Wind (m/s)**	**V-Wind (m/s)**	**Wangerooge**	**Juist**	**Terschelling**	**Caister-on-Sea**
1000 hPa	9.87 ± 3.19 (2.25 – 18.85)	79.5 ± 8.89 (53 – 99)	1.37 ± 6.04 (-17.2 – 15.6)	1.73 ± 4.84 (-12.0 – 14.9)	-1.76 ± 4.82 (-14.97 – 11.92)	-2.18 ± 5.10 (-13.17 – 14.64)	-2.01 ± 5.48 (-12.48 – 15.07)	-1.67 ± 5.86 (-14.59 – 16.39)
925 hPa	6.32 ± 4.30 (-2.75 – 19.45)	75.15 ± 18.93 (9 – 100)	4.18 ± 7.47 (-19.9 – 20.6)	1.80 ± 6.64 (-16.9 – 20.3)	-1.87 ± 6.61 (-20.6 – 16.82)	-4.31 ± 6.35 (-26.66 – 17.00)	-4.54 ± 6.71 (-24.90 – 18.88)	-4.45 ± 7.18 (-21.15 – 19.47)
850 hPa	3.02 ± 5.00 (-6.85 – 15.05)	64.15 ± 24.67 (0 – 100)	4.62 ± 7.16 (-18.5 – 23.0)	1.59 ± 6.54 (-19.60 – 18.00)	-1.67 ± 6.51 (-18.09 – 19.24)	-4.49 ± 6.16 (-28.13 – 13.14)	-4.83 ± 6.47 (-27.98 – 15.31)	-4.83 ± 6.89 (-25.73 – 17.48)
Spring					**St. Peter-Ording**	**Amrum**	**Blåvand**	**Maland**
1000 hPa	4.45 ± 3.11 (-3.85 – 17.05)	83.56 ± 8.52 (38 – 100)	0.76 ± 8.02 (-19.4 – 13)	0.11 ± 3.92 (-10.5 – 12)	0.76 ± 7.78 (-18.46 – 12.74)	0.48 ± 5.24 (-11.19 – 12.61)	0.15 ± 3.93 (-10.44 – 12.04)	0.07 ± 3.94 (-10.53 – 11.93)
925 hPa	0.77 ± 4.37 (-10.35 – 16.95)	84.05 ± 14.84 (30 – 100)	2.70 ± 10.19 (-22.7 – 20.5)	-0.39 ± 5.98 (-13.90 – 16.40)	2.49 ± 9.56 (-22.04 – 20.61)	1.01 ± 6.40 (-13.69 – 19.90)	-0.25 ± 5.88 (-14.05 – 16.94)	-0.54 ± 6.12 (-13.71 – 15.81)
850 hPa	-2.15 ± 4.70 (-13.95 – 12.65)	67.14 ± 25.74 (10 – 100)	3.70 ± 9.09 (-22.4 – 22.5)	-0.68 ± 5.73 (-13.00 – 13.70)	3.37 ± 8.61 (-20.43 – 24.41)	1.27 ± 6.12 (-12.66 – 20.00)	-0.48 ± 5.66 (-13.13 – 14.11)	-0.87 ± 5.82 (-12.84 – 13.48)

Supplementary Table 2. Variation in altitude-dependent weather conditions used as explanatory variables in correlation with arrival fuel load for each flight paths aligned with the coastal destination per season. Given are the mean ± standard deviation as well as the range (in brackets) of the variation in each variable.

**Autumn**	**Relative Humidity (%)**	**Tailwind components (m/s)**
Wangerooge	73.60 ± 21.99<br>(0-98)	0.06 ± 5.87<br>(-14.74-20.30)
Juist	77.89 ± 15.48<br>(7-100)	-1.62 ± 5.23<br>(-13.44-16.65)
Terschelling	78.15 ± 14.53<br>(7-100)	-1.69 ± 5.65<br>(-12.48-18.79)
Caister-on-Sea	78.36 ± 14.20<br>(7-100)	-1.65 ± 5.81<br>(-12.44-19.17)
**Spring**		
St. Peter-Ording	77.82 ± 22.17<br>(11-100)	3.96 ± 8.53<br>(-17.84-24.52)
Amrum	79.67 ± 20.21<br>(11-100)	2.56 ± 5.72<br>(-9.15-19.52)
Blåvand	79.97 ± 16.14<br>(21-100)	1.76 ± 4.95<br>(-10.38-17.28)
Mandal	80.41 ± 15.64<br>(21-98)	1.48 ± 5.01<br>(-10.53-15.81)

Chapter 4: Details to coastal destinations

Supplementary Table 3: Coordinates, flight distances and flight direction for selected coastal destination.

Season	Coastal destination	Flight distance (km)	Flight direction (°)
**Autumn**	Wangerooge, Germany (53.79°N, 7.90°E)	43	178
	Juist, Germany (53.68°N, 7.00°E)	80	226
	Terschelling, Netherlands (53.44°N, 5.49°E)	117	242
	Caister-on-Sea, United Kingdom (52.63°N, 1.74°E)	442	247
**Spring**	St. Peter-Ording, Germany (54.32°N, 8.60°E)	49	72
	Amrum, Germany (54.67°N, 8.31°E)	61	27
	Blåvand, Denmark (55.57°N, 8.09°E)	155	5
	Mandal, Norway (58.03° N, 7.51° E)	429	357

Chapter 5: Overview of average fuel-dependent flight range

In addition to the measurements of the main text, fat score (levels 0 to 8) was estimated for each blackbird according to Kaiser (1993). In this study, blackbirds only showed fat score levels from zero to five. A linear model (LM) was used to analyse absolute fuel loads (dependent variable) between different fat score levels (six-level explanatory factor: 0 to 5). Since the arrival fuel load (dependent variable) is given in relative proportions, we also used a generalised linear model with a binomial error distribution (GLM; family "quasibinomial") to correlate arrival fuel load (dependent variable) with fat score levels (six-level explanatory factor: 0 to 5).

Absolute and relative arrival fuel loads differed between the fat score levels (Supplementary Table 3), except for the comparison between fat score level "0" and "1". Here, CrI overlapped in term of absolute and relative arrival fuel loads; most likely due to the small sample size for level "0". All of the blackbirds classified in fat score level "5" had sufficient arrival fuel loads enabling them to fly between 446 km and 1,185 km in still air without further refuelling, theoretically allowing them to reach all possible coastal destinations. On the other hand, none of the blackbirds with a fat score of "0" would have been able to reach the next coastal destination in still air.

Supplementary Table 4: Fat score levels, absolute and (relative) arrival fuel load and potential flight range. We have described the estimated mean (in bold) and CrI (in brackets). Corresponding potential flight ranges (km) are given considering still air (groundspeed = 10 m/s).

Fat Score	Sample Size	Absolute fuel load (g)	Arrival fuel load (rel.)	Potential flight range (km)
0	6	**0.06**	**0.001**	**4**
		(-3.7 – /3.88)	(0 – 0.127)	
1	104	**1.69**	**0.019**	**68**
		(0.78 – 2.58)	(0.015 – 0.025)	
2	305	**4.89**	**0.055**	**193**
		(4.33 – 5.42)	(0.050 – 0.060)	
3	486	**9.37**	**0.104**	**356**
		(8.95 – 9.78)	(0.099 – 0.109)	
4	392	**14.93**	**0.165**	**549**
		(14.49 – 15.30)	(0.159 – 0.172)	
5	19	**23.24**	**0.251**	**806**
		(21.04 – 25.14)	(0.218 – 0.286)	

Kaiser A. A new multi-category classification of subcutaneous fat deposits of songbirds (Una Nueva Clasifcación, con Multi-categorías, para los Depósitos de Grasa en Aaves Canoras). J Field Ornithol. 1993;64:246-255.

Chapter 6: Excluded model parameters and additional results

Initially, the models analysing arrival fuel load between migrants caught after "unfavourable weather" nights and migrants caught otherwise (favourable weather") included the interaction between relative humidity and TWC, but as the interaction did not show an effect in any of our models, it was excluded from further analysis.

All models initially included year as a random factor; however, as the variance of this parameter had an effect of (close to) zero, we excluded it from our models. Time of day-effects were not considered as relative fuel load showed high variability during the entire day with coefficients of determination (R^2) close to zero in both seasons (spring: $R^2 = 0.0003$; autumn: $R^2 = 0.0007$), which is in accordance with Dierschke & Bindrich (2001).

Supplementary Table 5: Rate of successful flights (%) depending on the coastal destination under a) still air and b) individual experienced wind conditions. Data are given for all migrating blackbirds (n=1312), broken down by sex, age and season. For each group, sample size (n), mean values and 95% credible intervals (2.5% and 97.5%) are reported.

				Successful flights [%]							
**Season**	**Sex**	**Age**	**n**	**Wangerooge (43 km)**		**Juist (80 km)**		**Terschelling (177 km)**		**Caister-on-Sea (442 km)**	
				**a)**	**b)**	**a)**	**b)**	**a)**	**b)**	**a)**	**b)**
**Autumn**	Male	First-years	212	85	64	80	57	63	39	25	10
		Adults	219	97	79	95	73	89	52	52	14
	Female	First-years	249	88	67	83	59	66	38	25	11
		Adults	239	94	71	90	67	79	42	41	13
				**St. Peter-Ording (49 km)**		**Amrum (61 km)**		**Blåvand (155 km)**		**Mandal (429 km)**	
**Spring**	Male	Adults	200	94	83	89	77	82	68	42	34
	Female	Adults	193	89	75	85	70	75	54	30	20

Dierschke V, Bindrich F. Body condition of migrant passerines crossing a small ecological barrier. Vogelwarte. 2001;41:119-132.

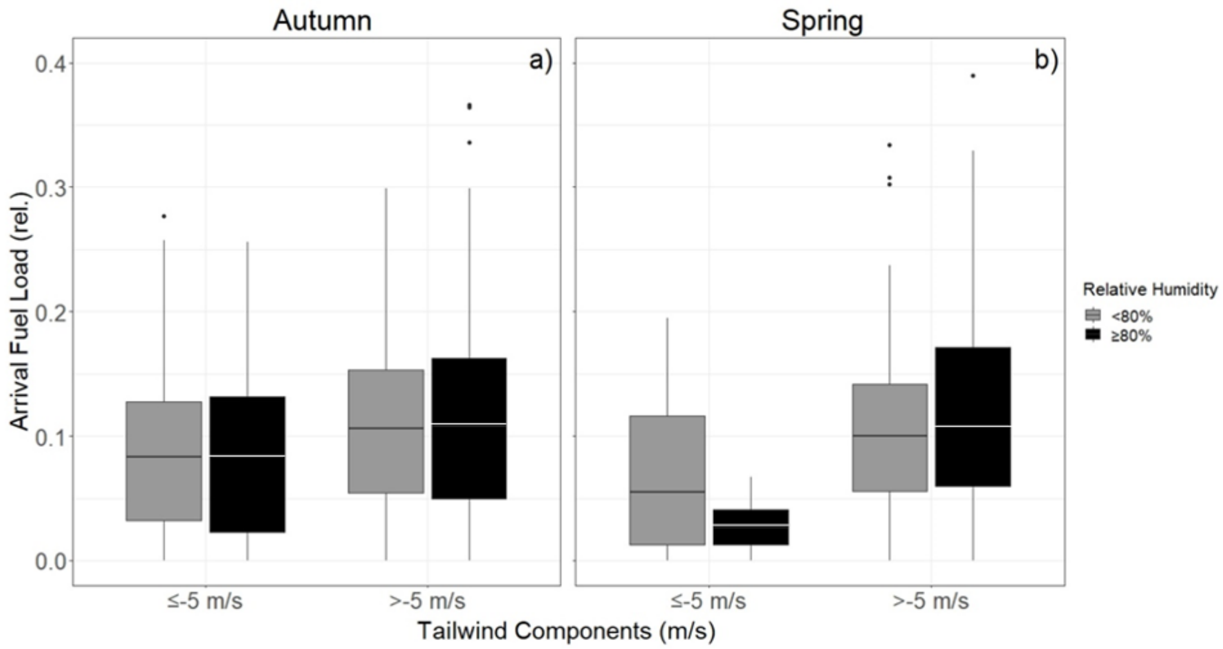

Fig. 2: Arrival fuel loads (relative values) of blackbirds stopping on Helgoland following nights with adverse (≤ -5 m/s) or favourable (> -5 m/s) winds and favourable (grey n-rhum) or unfavourable (black h-rhum) relative humidity conditions in autumn (a) and spring (b). The boxplots show 5, 25, 50, 75 and 95 percentiles as well as outliers. Exemplary weather values are given here for flight paths oriented towards the coastal destination Wangerooge (autumn) and St. Peter-Ording (spring). Sample sizes for a) ≤ -5 m/s = 115 (n-rhum) and 76 (h-rhum), > -5 m/s = 280 and 448; b) ≤ -5 m/s = 45 and 10, > -5 m/s = 111 and 227.

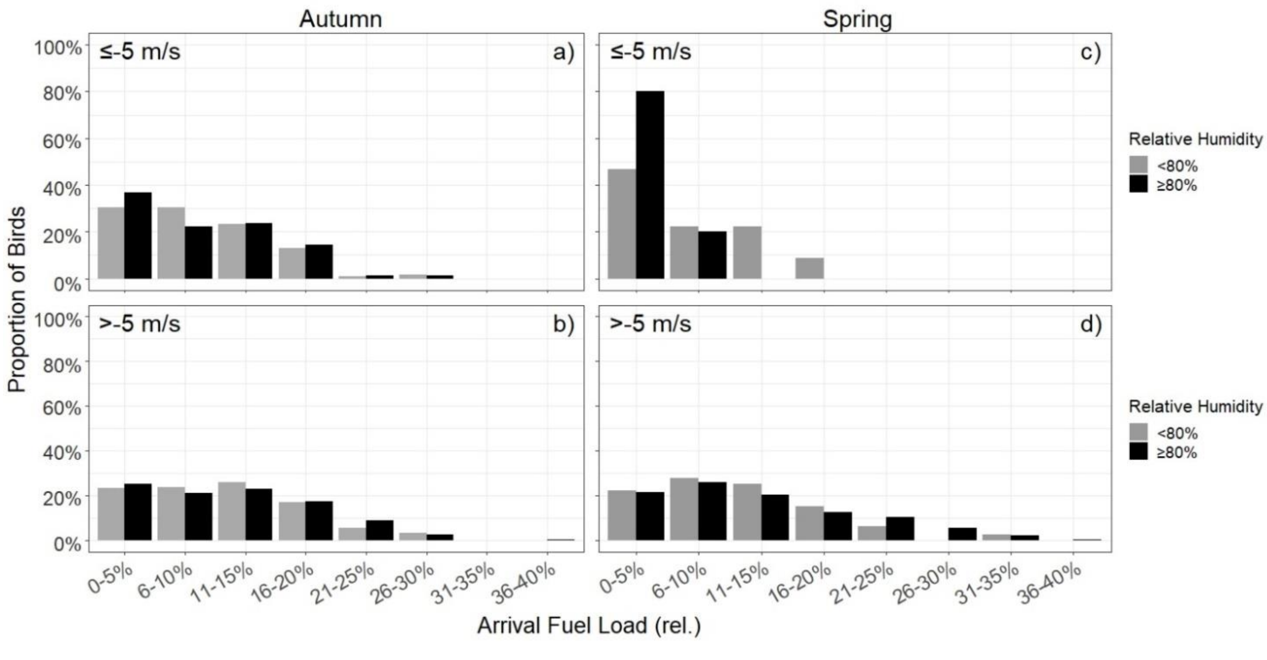

Fig. 3: Arrival fuel loads (relative values) of blackbirds caught on Helgoland during autumn (n=919; a, b) and spring (n=393; c, d) migration. Data is subdivided depending on adverse (≤ -5 m/s; A, C) and favourable (> -5 m/s; b, d) winds as well as relative humidity < 80% (n-rhum; grey bars) and ≥ 80% (h-rhum; black bars) experienced on the night before arrival. Birds were classified according to their arrival fuel load. Exemplarily, weather values are given for the flight paths towards Wangerooge (autumn) and St. Peter-Ording (spring). Sample sizes for a) 115 (n-rhum) and 76 (h-rhum) individuals, b) 280 and 448, c) 45 and 10, d) 111 and 227.
